# ACTL6A regulates follicle-stimulating hormone-driven glycolysis in ovarian cancer cells via PGK1

**DOI:** 10.1038/s41419-019-2050-y

**Published:** 2019-10-24

**Authors:** Jiawen Zhang, Jing Zhang, Yingze Wei, Qingxian Li, Qingying Wang

**Affiliations:** 1 Department of Obstetrics and Gynecology, Shanghai Tenth People’s Hospital, School of Medicine, Tongji University, Shanghai, China; 2 Department of Integrated Therapy, Shanghai Cancer Center, Fudan University, Shanghai, China; 3 Department of Oncology, Shanghai Medical College, Fudan University, Shanghai, China; 4 Department of Pathology, Nantong Tumor Hospital, Nantong, Jiangsu China; 5 Department of Gynaecology and Obstetrics, Putuo Hospital, Shanghai University of Traditional Chinese Medicine, Shanghai, China

**Keywords:** Cancer metabolism, Ovarian cancer

## Abstract

Enhanced glycolysis has been identified as a hallmark of cancer. As a novel oncogene, ACTL6A is aberrantly amplified in several types of human cancers and has been shown to regulate tumor growth and progression. However, the roles of ACTL6A in the development of ovarian cancer and the regulation of cancer glucose metabolism are mostly unknown. Here we show that ACTL6A is overexpressed in ovarian cancers compared with adjacent non-tumor tissues, and that ACTL6A overexpression correlates with poor prognosis. Silencing of ACTL6A in vitro inhibits proliferation, clonal growth, and migration, and decreases glucose utilization, lactate production, and pyruvate levels of ovarian cancer cells. We found a positive correlation between ACTL6A and PGK1 expression in ovarian cancer tissues. Enforced ACTL6A expression increased PGK1 expression, whereas knockdown of ACTL6A had the opposite effect. Altered ACTL6A expression inhibits the tumorigenicity of ovarian cancer cells in vivo by downregulating PGK1. In addition, the expression of ACTL6A is regulated by follicle-stimulating hormone (FSH) stimulation via PI3K/AKT pathway. Importantly, ACTL6A regulates FSH-enhanced glycolysis in ovarian cancer. Taken together, our findings highlight the critical role of ACTL6A in ovarian cancer development and identify its contribution to glucose metabolism of cancer cells.

## Introduction

Epithelial ovarian cancer (EOC) is generally diagnosed at an advanced stage and is the most lethal gynecological cancer. In 2018, ~295,000 new EOC cases and 185,000 cancer deaths are projected to occur globally^1^. Despite various advances in early detection strategies and systematic therapies for EOC made over the past decades, about 75% of patients are diagnosed with advanced-stage disease and the 5-year survival rate still remains low^2^. The need to identify potential targets for therapeutic intervention to improve EOC patient outcomes is urgent.

Mounting evidence indicates that cancer cells have enhanced glucose uptake and lactate accumulation to meet the increased energy demands of tumor growth and aggressiveness^3^. This phenomenon is commonly referred to as the Warburg effect and is supposed to be the most fundamental metabolic changes in the process of tumorigenesis^4^. The dependence of ovarian cancer cell proliferation and progression on their glycolytic property has been documented and several enzymes in the glycolytic pathway have also been identified as promising therapeutic targets for anticancer intervention^5–7^. Phosphoglycerate kinase 1 (PGK1) is the first ATP-generating enzyme in the glycolytic pathway, which catalyzes the conversion of 1,3-diphosphoglycerate to 3-phosphoglycerate^8^. In the context of cancer, PGK1 has been reported to be dysregulated in several tumor types^9–12^. However, the regulation of PGK1 in ovarian cancer cells and the underlying molecular mechanisms are still poorly understood.

SWI/SNF complexes (also known as BAF complex) are evolutionarily conserved multi-subunit protein complexes that mediate chromatin-remodeling processes, which is crucial for the regulation of gene expression^13^. In particular, the KlSnf2 subunit of SWI/SNF complexes is implicated in the glucose signaling to reprogram glucose metabolism by controlling Sms1 degradation in kluyveromyces lactis^14^. In human lung cancer, the SWI/SNF catalytic subunit BRG1 directly regulates the expression of MAX and MAX requires BRG1 to upregulate MYC targets including glycolysis-related genes^15^. These studies indicate that SWI/SNF complexes participate in the regulation of glycolysis. Increasing evidence has suggested that *ACTL6A* (also known as *Baf53a* or *Arp4*), a gene encoding a component of the SWI/SNF complex, functions as an oncogene in various cancer types^16–20^. As examples, ACTL6A was identified as an activator of Epithelial-to-mesenchymal transition (EMT)^16,21,22^. Recent studies also reported that ACTL6A interacts with p63 to facilitate cancer cell proliferation through Hippo-YAP pathway and binds to p53 to promote differentiation via the Sox2/Notch1 signaling^18,23^. Nevertheless, the role of ACTL6A in glycolysis and tumorigenicity of ovarian cancer remains unclear.

According to the gonadotropin hypothesis concerning the development of ovarian cancer, high level of follicle-stimulating hormone (FSH) has a stimulatory effect on ovarian surface epithelial cells and leads to malignant transformation by altering certain signaling pathways^24–27^. Although one recent study suggests that FSH may promote glycolysis in EOC^28^, little is currently known regarding the exact mechanism of FSH-induced glycolysis. In the current work, we uncover a unique role of ACTL6A that regulates glycolysis in ovarian cancer. Our study further reveals that silencing of ACTL6A attenuates FSH-driven ovarian cancer glycolysis by downregulating PGK1 expression. Therefore, our results provide a molecular mechanism underlying antitumor activity by targeting ACTL6A and lay a foundation to improve the clinical outcome of ovarian cancer patients.

## Materials and methods

### Cell lines and culture

NIH:OVCAR-3, SKOV3, and Hey were purchased from the American Type Culture Collection (Manassas, VA, USA). HO8910 and HEK293T were purchased from the Type Culture Collection of the Chinese Academy of Sciences (Shanghai, China). Mycoplasma infection was not found in all cell lines. All cells were routinely cultured in Dulbecco’s modified Eagle’s medium (high glucose, Invitrogen, USA) at 37 °C in a humidified atmosphere incubator containing 5% CO_2_. Media was supplemented with 10% fetal bovine serum (Invitrogen, Carlsbad, CA) and 1% penicillin/streptomycin.

### Reagents

ACTL6A (ab131272) and FSH receptor (FSHR) (ab75200) antibodies were from Abcam (Cambridge, MA, USA). ACTL6A (sc-137062) mouse monoclonal antibody was from Santa Cruz (Dallas, TX, USA). AKT (#9272) and Phospho-AKT (Ser473) (#4060) antibodies were from Cell Signaling Technology (Danvers, MA, USA). PGK1 (17811-1-AP), GAPDH (10494-1-AP), and secondary antibodies were from Proteintech (Wuhan, China). FSH (869001) was from Merck (Darmstadt, Germany). LY294002 (S1105) and MK2206 (S1078) were from Selleck (Houston, USA). MTT (3-(4,5-dimethylthiazol-2-yl)-2,5-diphenyltetrazolium bromide) kit (C0009) was obtained from Beyotime Biotech, Inc. (Shanghai, China). Transwell® Polycarbonate Membrane (#3422) was obtained from Corning (NY, USA).

### Human tissue microarray and immunohistochemical analysis

The study was approved by the Institutional Ethics Committee of Tongji University Affiliated Shanghai Tenth People’s Hospital. The tissue microarray of ovarian cancer including 80 tumor samples and 10 paired adjacent normal tissues were purchased from Shanghai Zuocheng Biotech (Shanghai, China). Pathological diagnoses of the samples were confirmed based on the World Health Organization classification by independent pathologists. Immunohistochemical (IHC) analysis of ACTL6A and PGK1 protein expression was performed as previously reported^29^. The staining intensity was scored as follows: score = 0, negative; score = 1, weak; score = 2, moderate; score = 3, strong. The staining extent was scored as follows: score = 0, no positive cells; score = 1, ≤25% positive cells; score = 2, 26–49% positive cells; score = 3, 50–74% positive cells; and score = 4, ≥75% positive cells. Finally, the immunoreactivity score was calculated by the intensity score multiplying the extent score, resulting in a negative (0) level, a low (1–4) level, a medium (5–8) level, or a high (9–12) level values for each specimen.

### Data mining

Gene expression data available at The Cancer Genome Atlas (TCGA) database was assessed using the cBioPortal (http://cbioportal.org), to investigate the genomic and gene expression profiling of ACTL6A and PIK3CA, as well as the correlation of ACTL6A expression with glycolysis-related gene expression in ovarian serous cystadenocarcinoma^30,31^. The publicly available Oncomine database (https://www.oncomine.org/) and the Gene Expression Omnibus (GEO) database were used to compare the expression of ACTL6A in ovarian cancer tissues and normal tissues. GSE69428 evaluated the differential gene expression between high-grade serous ovarian cancer and paired normal oviduct samples from ten independent patients. GSE10971 compared gene expression profiles of laser capture microdissected non-malignant fallopian tube epithelium (FTE) from 12 normal control patient and 13 high-grade tubal and ovarian serous carcinomas. GSE28979 assessed gene expression patterns in three normal mouse fallopian tube oviduct and three early tumors from fallopian tubes of Dicer/PTEN knockout mice. GEO database was also used to provide insight into the biological function and regulation of ACTL6A. GSE88831 investigated the alterations in gene expression after treatment with short hairpin RNA (shRNA) for ACTL6A in head and neck squamous cell carcinoma (HNSCC) cell line FaDU. GSE120991 analyzed the changes in gene expression when HaCat cell line was treated with phosphatidyl inositol 3-kinase (PI3K) inhibitor LY294002. GSE69893 analyzed the changes in gene expression when MCF7 cell line was treated with AKT antagonist AZD5363. Biological processes related to the activities of ACTL6A in ovarian cancer were evaluated by GSEA v3.0 software^32,33^. Processing of the data was according to the guidelines of these databases.

### Plasmid transfection and gene silencing

The entire coding region of ACTL6A was cloned into the pcDNA3.1-Flag or pcDNA3.1-HA vectors. The plasmid vectors were transfected into ovarian cancer cells with Lipofectamine 3000 (Invitrogen, USA) according to the manufacturer’s instructions. The shRNA against ACTL6A was cloned into the pLKO.1 vector and infected cells were selected in puromycin. The small interfering RNAs (siRNAs) against PGK1, c-Myc, and FSHR were purchased from GenePharma (Shanghai, China). Cells were transfected with siRNA oligonucleotides using Lipofectamine 2000 (Invitrogen, USA). The sequences for shRNA and siRNA are listed in Supplementary Table S1.

### Real-time quantitative PCR

Total RNA extraction from ovarian cancer cells was performed using the RNAiso Plus (9108, Takara) according to the manufacturer’s directions. One microgram of total RNA was reverse transcribed using PrimeScript™ Reverse Transcriptase (2690S, Takara) to synthesize cDNA samples. Quantitative PCR was performed with SYBR qPCR Master Mix (TOYOBO) and quantified by the CFX Real-Time PCR Detection System (Bio-Rad). The sequences of the primers: PGK1, sense strand 5′-GAACAAGGTTAAAGCCGAGCC-3′, antisense strand 5′-GTGGCAGATTGACTCCTACCA-3′; β-actin, sense strand 5′-CATGTACGTTGCTATCCAGGC-3′ and antisense strand 5′-CTCCTTAATGTCACGCACGAT-3′.

### Cell proliferation and colony-formation assay

For cell proliferation assay, a total of 1000 cells per well were seeded in 96-well plates. At the end of treatment, MTT solution (10 μl of 10 mg/ml MTT in phosphate-buffered saline (PBS)) was added to each well. After 4 h of incubation at 37 °C, 200 μl dimethyl sulfoxide was added and absorbance values were then measured at an absorbance wavelength of 490 nm using a microplate reader. For colony-formation assay, cells were seeded in 12-well plates at an initial cell density of 200 cells per well and were grown for 10–14 days. Colonies were stained with crystal violet for 30 min at room temperature after fixation with 4% paraformaldehyde. Plates were photographed after extensive washing and drying. Each experiment was repeated at least three times independently.

### Cell migration

Transwell assay was performed as previously described^26^. Briefly, cells (20,000 cell/chamber) were seeded on top of Transwell chambers (Corning) after incubated in serum-free medium for 24 h. Medium was supplemented with serum-free medium alone or with added FSH in the upper chamber, and the lower chamber was filled with 10% fetal bovine seum as a chemoattractant. After incubation for 24 h, cells were fixed with 4% paraformaldehyde for 20 min, followed by staining with crystal violet for 30 min, and washed three times with PBS. Each experiment was repeated at least three times independently.

### Measurement of glucose uptake

Glucose uptake was determined using Glucose Uptake Colorimetric Assay Kit (ab136955, Abcam, Cambridge, MA, USA) according to the manufacturer’s instruction. Measurements were performed for at least five replicates.

### Measurement of lactate production

Lactate production in the culture medium was determined using l-Lactate Assay kit (ab65331, Abcam, Cambridge, MA, USA) according to the manufacturer’s instruction. Measurements were performed for at least five replicates.

### Measurement of pyruvate level

Pyruvate level was determined using Pyruvate Assay Kit (ab65342, Abcam, Cambridge, MA, USA) according to the manufacturer’s instruction. Measurements were performed for at least five replicates.

### FSH treatment

For immunoblot analysis, ovarian cancer cells were stimulated with FSH for the indicated periods of time (0, 24, 48, and 72 h) at the indicated concentrations (0, 25, 50, and 100 mIU/ml). For functional experiments, ovarian cancer cells were stimulated with 50 mIU/ml FSH for 48 h.

### Tumor xenografts

Female nude mice were purchased from Shanghai SLAC laboratory Animal Co., Ltd (Shanghai, China). The experimental procedures were approved by the ethics committee of Tongji University Affiliated Shanghai Tenth People’s Hospital and were conducted in accordance with the guidelines of the Institutional Animal Care and Use Committee. OVCAR-3 cells (5 × 10^6^) infected with control shRNA (*n* = 6) or shACTL6A (*n* = 6) were subcutaneously injected into the dorsal flank of nude mice respectively. After 10 days, tumor volume was measured every 4 days and was calculated using the equation (width^2^ × length × 0.5). On the 30th day, mice were killed and tumors were excised. After being photographed and weighed, tumors were lysed for western blotting analyses or embedded into paraffin for IHC.

### Immunoprecipitation and western blotting

To detect endogenous protein interactions, cells were lysed in co-immunoprecipitation lysis buffer (50 mM Tris-Cl, pH 7.4, 10% glycerol, 150 mM NaCl, 1 mM EDTA, 0.5% Nonidet P-40, and proteinase inhibitors). After 30 min, the cell lysates were cleared by centrifugation and then incubated with 3 μl anti-ACTL6A antibody and 15 μl protein A/G beads at 4 °C overnight. After extensive wash of the beads, the captured proteins were boiled at 100 °C for 10 min. Western blotting analysis was performed as previously described^29^.

### Statistical analysis

The data were presented as mean ± SD. Student’s *t*-tests were used to determine statistical significance of differences between experimental groups. For correlation analysis, Pearson’s correlation analysis and Robust correlation analysis from the Robust Base package in R were used. Survival curves were estimated by the Kaplan–Meier method and were compared using the log-rank test. All tests were two-sided and *p*-value < 0.05 was considered significant. Graphs were created with GraphPad Prism (Version 6.01, GraphPad Software, Inc., USA).

## Results

### The *ACTL6A* gene is frequently amplified in ovarian cancer

**Fig. 1:**
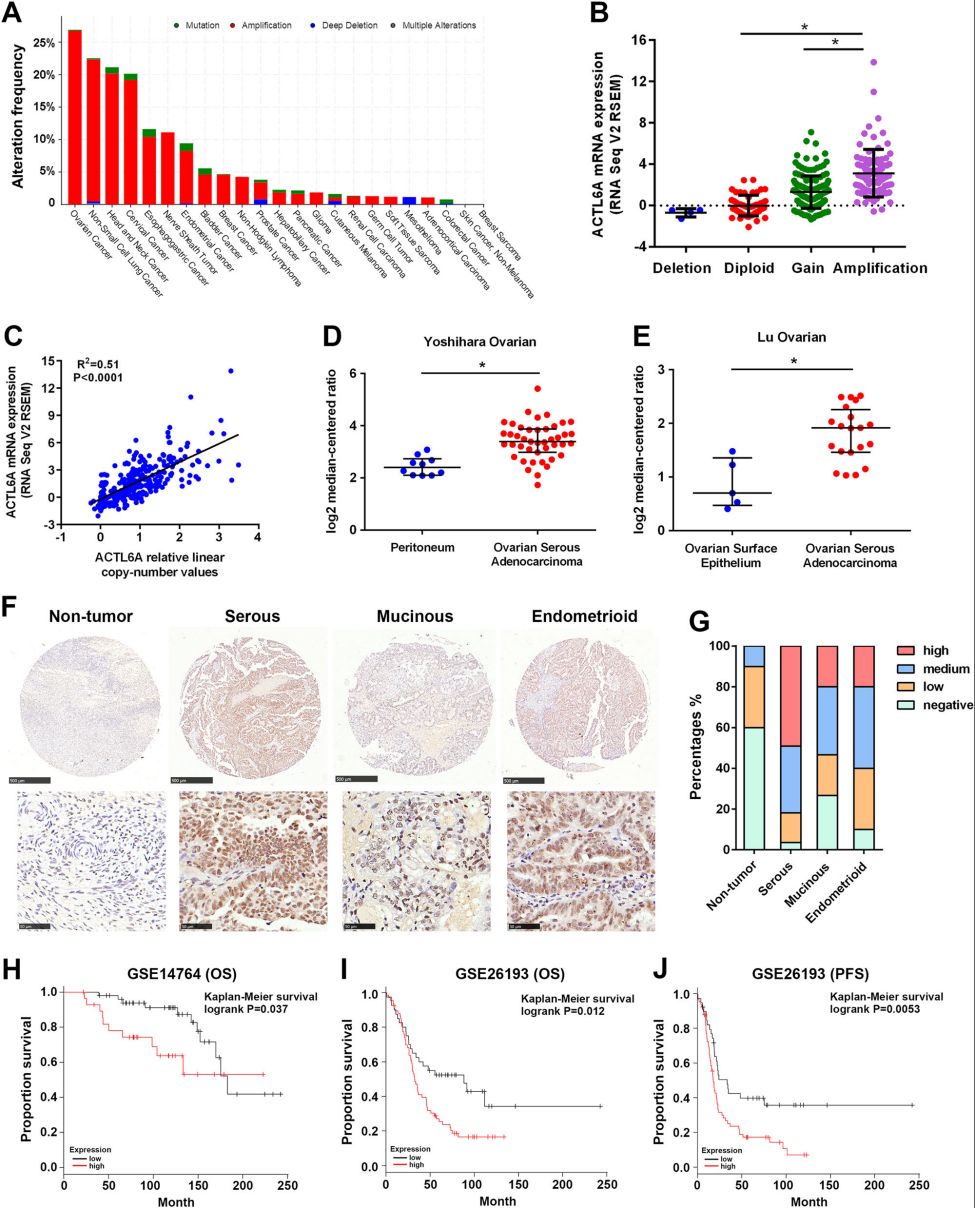
The *ACTL6A* gene is frequently amplified in ovarian cancer. **a** Genomic profiling of ACTL6A across human cancers determined by cBioPortal analysis (http://www.cbioportal.org/) of TCGA databases. **b** Positive correlation of ACTL6A mRNA expression with its copy number alteration in ovarian cancer from TCGA databases. *p < 0.01. **c** Scatterplots of ACTL6A mRNA expression vs. copy number in ovarian cancer from TCGA databases. **d**, **e** Analysis of ACTL6A expression in ovarian cancer and peritoneum or ovarian surface epithelium in two independent datasets available at Oncomine (https://www.oncomine.org/). *p < 0.01. **f** Representative pictures of ACTL6A IHC staining in ovarian cancer tissues and adjacent non-tumor tissues. Scale bar = 500 μm (upper) and 50 μm (lower). **g** The percentage of ACTL6A staining in adjacent non-tumor tissues (*n* = 10) and different subtypes of EOC tissues (serous, *n* = 55; mucinous, *n* = 15; endometrioid, *n* = 10). **h**, **i** Kaplan–Meier overall survival (OS) curves in ACTL6A-high and low expression ovarian cancer cases from GSE14764 and GSE26193. **j** Kaplan–Meier progression-free survival (PFS) curves in ACTL6A-high and low expression ovarian cancer cases from GSE26193

Our analysis of genomic profiling of several cancer types in TCGA demonstrated that *ACTL6A* gene is amplified in 26.73% of ovarian cancer (Fig. 1a) and the amplification is the most common genetic event of ACTL6A in ovarian cancer (Fig. 1b). Copy number of *ACTL6A* is significantly correlated with its mRNA expression (*R*^2^ = 0.51, *p* < 0.0001; Fig. 1c). Oncomine database revealed that ACTL6A is more highly expressed in ovarian cancer tissues than in ovarian surface epithelium or peritoneum tissues (Fig. 1d, e). Given growing evidence implicating the distal FTE as a common source for EOC, we analyzed clinically annotated expression data from GEO databases. The expression of ACTL6A is higher in EOC than in normal oviduct or FTE (GSE69428 and GSE10971; Supplementary Fig. S1a, b). Moreover, in comparison with normal mouse fallopian tube oviduct, early tumors from the fallopian tubes of Dicer/PTEN knockout mice have 1.77-fold higher expression levels of ACTL6A (GSE28979; Supplementary Fig. S1c).

In the IHC analysis using a tissue microarray of ovarian cancer including 80 tumor samples and 10 adjacent normal tissues, nearly three-quarters (*n* = 59, 73.8%) of tumor samples had a medium to high IHC scores of ACTL6A, whereas little positive staining was detected in the adjacent non-tumor tissues (Fig. 1f, g). Positive staining was also observed in different pathological subtypes of EOC. Serous carcinomas showed strong positive staining, followed by endometrioid adenocarcinomas and mucinous carcinomas (Fig. 1f, g). Importantly, quantitative analysis for Kaplan–Meier survival curves in GSE14764 and GSE26193 cohorts showed significant correlation between high ACTL6A expression and poor overall survival in ovarian cancer patients (Fig. 1h–j)^34^. Collectively, these data suggest that high expression of ACTL6A may be related to ovarian tumorigenesis.

### The effect of ACTL6A on ovarian cancer phenotype

**Fig. 2:**
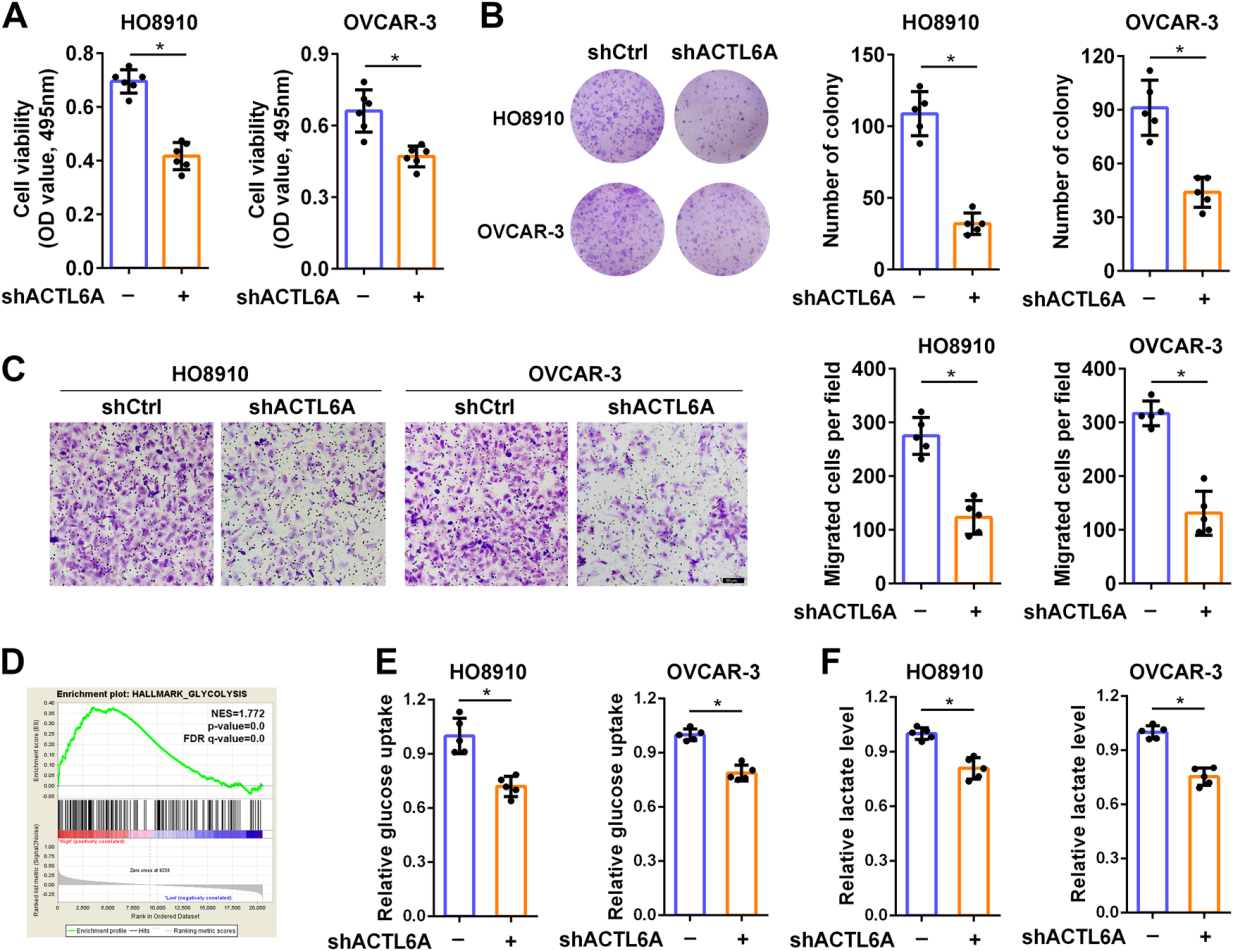
The effect of ACTL6A on ovarian cancer phenotype. **a** MTT assay of HO8910 and OVCAR-3 cells transfected with shCtrl or shACTL6A. **b** Colony-formation assay of HO8910 and OVCAR-3 cells transfected with shCtrl or shACTL6A. **c** Cell migration assay of HO8910 and OVCAR-3 cells transfected with shCtrl or shACTL6A performed in Transwell chamber followed by crystal violet staining. The number of cells per field was counted. Scale bar = 50 μm. **d** GSEA analysis showed that the gene set of HALLMARK_GLYCOLYSIS was enriched in the ACTL6A-high group. FDR, false discovery rate; NES, normalized enrichment score. **e**, **f** Glucose uptake (**e**) and lactate production (**f**) were measured in HO8910 and OVCAR-3 cells stably transfected with shCtrl or shACTL6A. *p < 0.01

It has been reported that ACTL6A plays critical roles in varied cancer phenotypes. Therefore, we employed lentivirus-mediated shRNA to silence endogenous ACTL6A, to establish the role of ACTL6A in ovarian cancer cell phenotypes. We first investigated the effect of ACTL6A on ovarian cancer cell proliferation by MTT and colony-formation assay. Knockdown of ACTL6A significantly decreased cell growth in HO8910 and OVCAR-3 cells (Fig. 2a). Compared with the control group, significant reduction in the number of colonies in shACTL6A group was also observed (Fig. 2b). Then we performed Transwell migration studies to examine whether ACTL6A affects the cell motility in ovarian cancer. An analogous decrease in migration was observed in shACTL6A cells when compared with controls (Fig. 2c). To investigate the potential biological pathways of ACTL6A implicated in ovarian tumorigenesis, GSEA was performed using TCGA data for 307 samples from ovarian cancer. Gene sets of HALLMARK_GLYCOLYSIS (NES = 1.772, *p* < 0.01), REACTOME_GLYCOLYSIS (NES = 1.263, *p* < 0.01), and KEGG_GLYCOLYSIS_GLUCONEOGENESIS (NES = 1.476, *p* < 0.01) were highly enriched in ACTL6A-high group (Fig. 2d and Supplementary Fig. S2a, b). This led us to explore the role of ACTL6A in the control of glycolysis. We detected decreased glucose uptake (Fig. 2e), lactate production (Fig. 2f), and pyruvate level (Supplementary Fig. S2c) in HO8910 and OVCAR-3 cells transfected with shACTL6A. These results indicated that ACTL6A affects ovarian cancer cell growth, mobility, and glycolysis, the three pivotal hallmarks of cancer^35^.

### ACTL6A promotes glycolysis through upregulation of PGK1

**Fig. 3:**
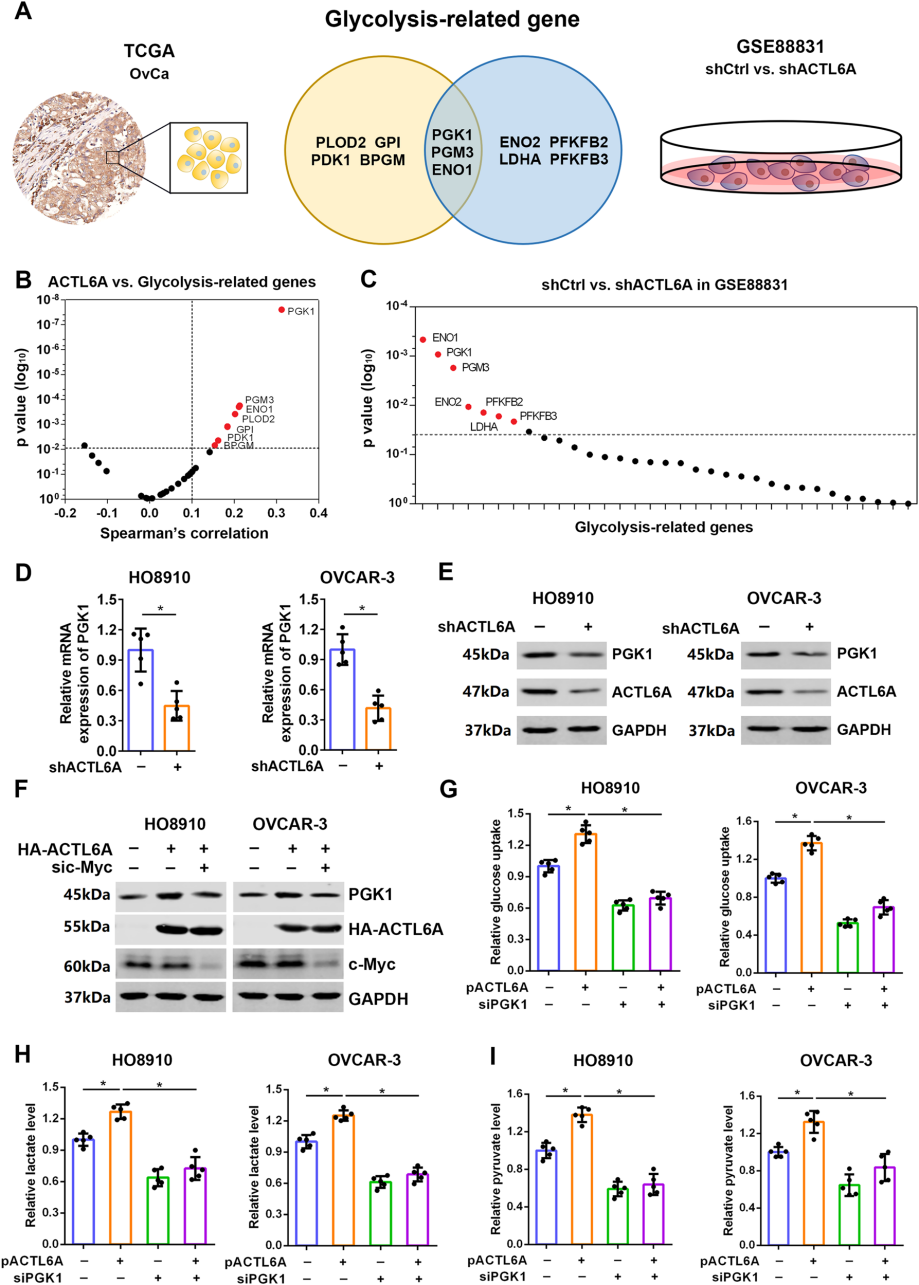
ACTL6A promotes glycolysis through upregulation of PGK1. **a** Venn diagram showing differentially expressed glycolysis-related genes in TCGA and GEO database. **b** The correlation between the expression of ACTL6A and glycolysis-related genes based on TCGA. **c** The expression alterations of glycolysis-related genes in GSE88831 database (shCtrl vs. shACTL6A). **d** qRT-PCR analysis of PGK1 mRNA expression in HO8910 and OVCAR-3 cells transfected with shCtrl or shACTL6A. **e** Western blot analysis of PGK1 protein level in HO8910 and OVCAR-3 cells transfected with shCtrl or shACTL6A. **f**–**h** Glucose uptake (**f**), lactate production (**g**), and pyruvate level (**h**) were measured in HO8910 and OVCAR-3 cells transfected with ACTL6A expression plasmid and PGK1 siRNA as indicated. *p < 0.01

To address whether ACTL6A could impact the expression of glycolysis-related genes in ovarian cancer, we analyzed the correlation between the expression of ACTL6A and glycolysis-related genes based on TCGA and interrogated the expression data that were deposited in GEO database (GSE88831: HNSCC cell line FaDU, shCtrl vs. shACTL6A) (Fig. 3a). As expected, ACTL6A expression correlated with several glycolysis-related genes, including *PGK1*, *PGM3*, *ENO1*, *PLOD2*, *GPI*, and *PDK1* (Fig. 3b and Supplementary Table S2). In line with this, among all the expression alterations of glycolysis-related genes in GSE88831 database, *PGK1*, *ENO1*, and *PGM3* were downregulated in shACTL6A cells (Fig. 3c and Supplementary Table S3). In view of *PGK1* was the most altered gene, we chose *PGK1* as the target gene and try to investigate whether ACTL6A-enhanced glycolysis in ovarian cancer was dependent upon PGK1. We next identified these findings using reverse-transcriptase quantitative PCR and western blotting, which demonstrated that the mRNA and protein level of PGK1 were significantly lower in shACTL6A cells than those in control cells (Fig. 3d, e and Supplementary Fig. S3a), whereas the protein level of PGK1 was upregulated in the cells transfected with ACTL6A expression plasmid (Supplementary Fig. S3b). Next, we investigated the mechanism of ACTL6A-upregulated PGK1. On the basis of a previous study on the role of ACTL6A in c-Myc oncogenic activity^36^, we determined that ACTL6A interacted with c-Myc in ovarian cancer cell OVCAR-3, but not PGK1 (Supplementary Fig. S3c); the silencing of c-Myc significantly inhibited ACTL6A-induced PGK1 (Fig. 3f and Supplementary Fig. S3d). Furthermore, in support of the involvement of PGK1 in ACTL6A-enhanced glycolysis, knockdown of PGK1 markedly reversed the glucose uptake (Fig. 3g), lactate production (Fig. 3h), and pyruvate level (Fig. 3i) of HO8910 and OVCAR-3 cells, which were upregulated by overexpression of ACTL6A. Therefore, we definitively prove that ACTL6A could regulate glycolysis by impacting PGK1 expression.

### Association of ACTL6A with PGK1 expression in ovarian cancer

**Fig. 4:**
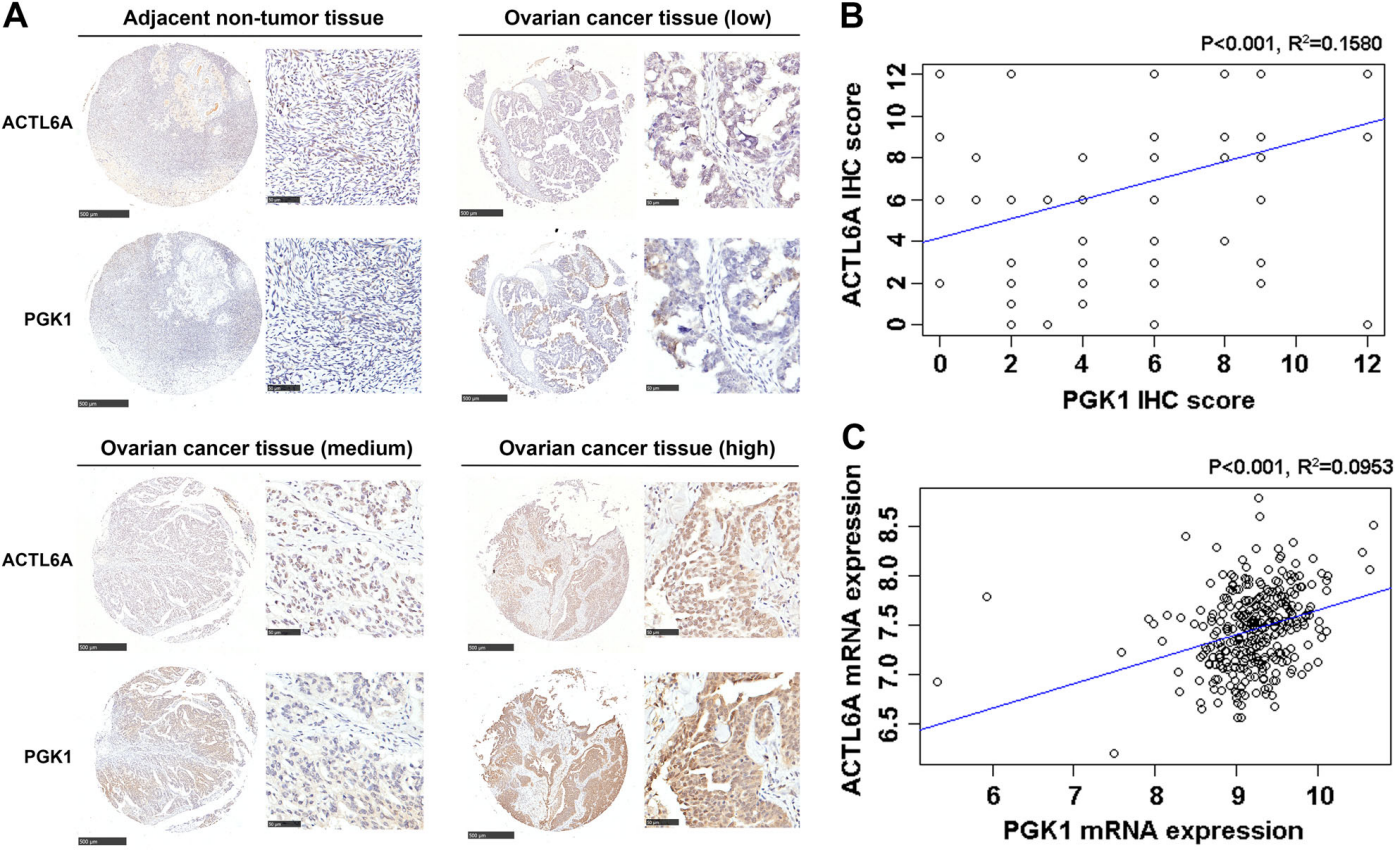
Association of ACTL6A with PGK1 expression in ovarian cancer. **a** IHC staining of ovarian cancer and adjacent non-tumor tissues for ACTL6A and PGK1. Shown are representative photographs of ACTL6A and PGK1 staining in four cases. Scale bar = 500 μm (left) and 50 μm (right). **b** Positive correlation of ACTL6A IHC score with PGK1 IHC score (*R*^2^ = 0.1580, *p* < 0.001). **c** Positive correlation of ACTL6A expression with PGK1 expression based on TCGA database (*R*^2^ = 0.0953, *p* < 0.001)

Next, we evaluated the relationship between ACTL6A and PGK1 in ovarian cancer tissues by IHC analysis. The protein level of ACTL6A was positively correlated with PGK1 at a statistically significant level (*R*^2^ = 0.1580, *p* < 0.001; Fig. 4a, b). Consistent with the protein level, the mRNA expression of ACLT6A was likewise positively correlated with PGK1 in ovarian cancer cohort from TCGA (*R*^2^ = 0.0953, *p* < 0.001; Fig. 4c). These data provided clinical evidence that overexpression of ACTL6A is associated with increased PGK1 expression.

### Silencing of ACTL6A inhibits the tumorigenicity of ovarian cancer cells in vivo

**Fig. 5:**
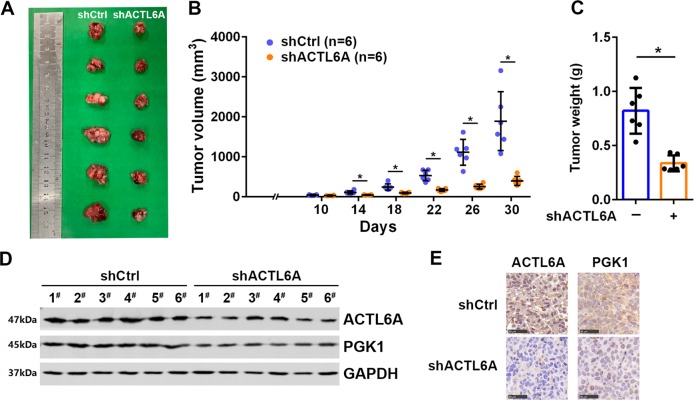
Silencing of ACTL6A inhibits the tumorigenicity of ovarian cancer cells in vivo. **a**, **b** Tumor formation in mice transplanted with OVCAR-3 cell expressing control or ACTL6A shRNAs. Tumor volumes were measured on indicated days. **c** Tumor weights are shown. **d** Western blot analysis of ACTL6A and PGK1 expression in xenograft. **e** IHC staining of ACTL6A and PGK1 in xenografts. Scale bar = 50 μm

The data above present that ACTL6A regulates glycolysis to promote ovarian cancer. To further support this model, we injected shCtrl or shACTL6A cells into the dorsal flank of nude mice subcutaneously and monitored tumor growth. The tumors derived from shACTL6A cells developed at a much slower rate than the outgrowths of shCtrl cells (Fig. 5a, b). On the 30th day, the mice were killed and the tumor tissues were collected for further analysis. We found that ACTL6A knockdown caused the decrease of tumor weight vs. control group (Fig. 5c). To confirm the impact of ACTL6A on PGK1, we investigated the expression of ACTL6A and PGK1 in xenografts by western blotting analyses and IHC staining. As shown in Fig. 5d, e, the protein level of PGK1 was lower in tumors derived from shACTL6A cells as compared with those from shCtrl cells, supporting the notion that ACTL6A functions as an upstream regulator of PGK1.

### ACTL6A is required for FSH-induced ovarian cancer cell glycolysis

**Fig. 6:**
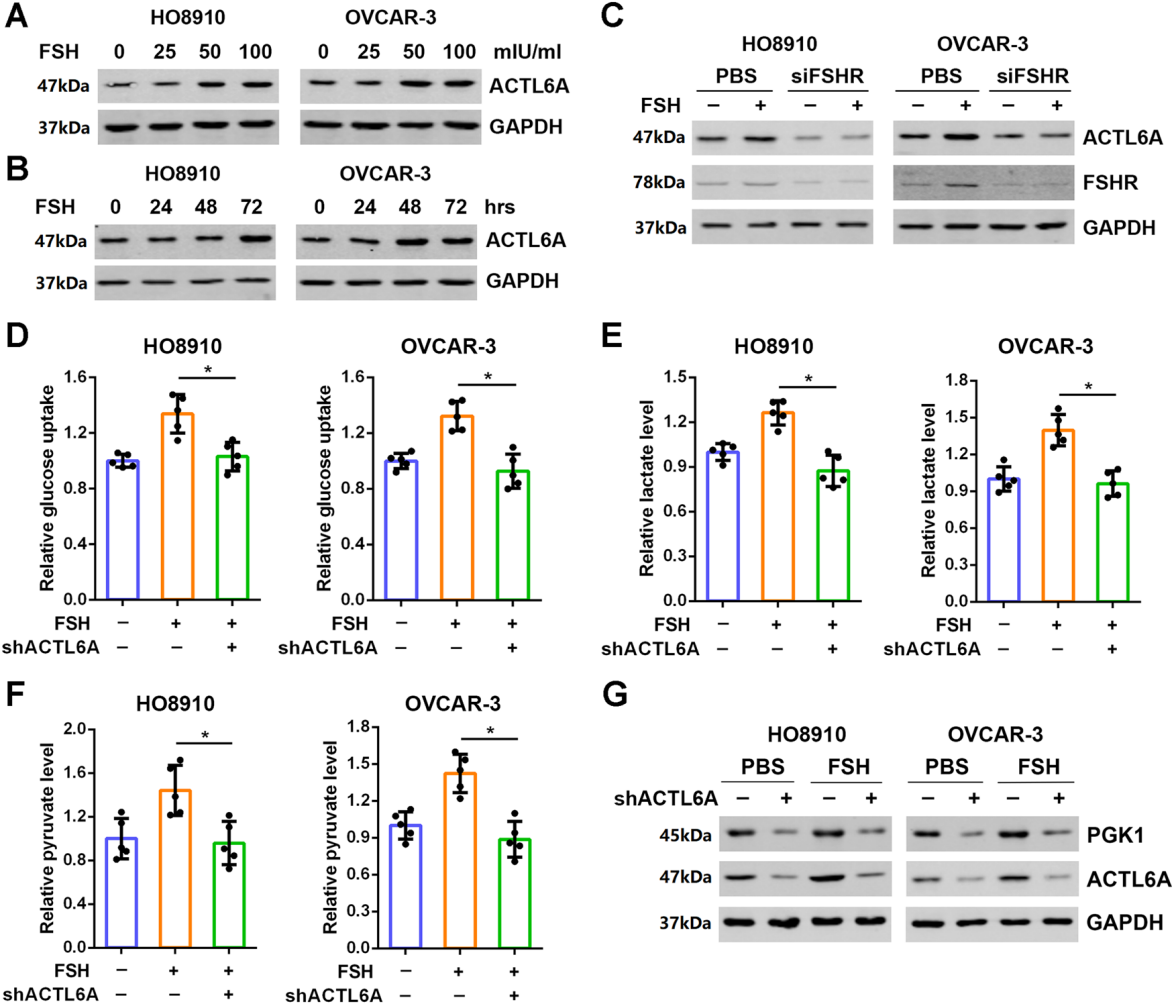
ACTL6A is required for FSH-induced ovarian cancer cell glycolysis. **a**, **b** Treatment with FSH upregulates ACTL6A expression in a dose- (**a**) and time-dependent (**b**) manner in HO8910 and OVCAR-3 cells. **c** Knockdown of FSHR blocks FSH-enhanced ACTL6A expression. **d**–**f** Glucose uptake (**d**), lactate production (**e**), and pyruvate level (**f**) were measured in HO8910 and OVCAR-3 cells stimulated by FSH with or without shACTL6A as indicated. *p < 0.01. **g** Knockdown of ACLT6A eliminates FSH-induced PGK1 expression

As a risk factor for oncogenesis of ovarian cancer, FSH has been shown to promote glycolysis^28^. Thus, we asked whether ACTL6A mediates FSH-regulated glycolysis. We found that stimulation of HO8910 and OVCAR-3 cells with FSH upregulated ACTL6A expression in a dose- and time-dependent manner (Fig. 6a, b). Moreover, knockdown of FSHR reduced ACTL6A protein level, whereas FSH-upregulated ACTL6A was also blocked by the silencing of FSHR (Fig. 6c). We further determined the role of ACTL6A in FSH-induced glycolysis. As shown in Fig. 6d–f, enhancement of glucose uptake, lactate production, and pyruvate levels stimulated by FSH was abolished by ACTL6A silencing. Subsequently, the increased cell growth, colony formation and cell motility induced by FSH could also be suppressed by ACTL6A knockdown (Supplementary Fig. S4a–c). These results demonstrate that ACTL6A is required for FSH carcinogenesis in ovarian cancer. In addition, we attempted to test whether PGK1 is involved in FSH-ACTL6A-glycolysis pathway. Western blotting analysis showed that stimulation of FSH enhanced PGK1 expression in ovarian cancer cells, which was further eliminated by downregulation of ACTL6A (Fig. 6g).

### FSH upregulation of ACTL6A and PGK1 depends on PI3K/AKT signaling

**Fig. 7:**
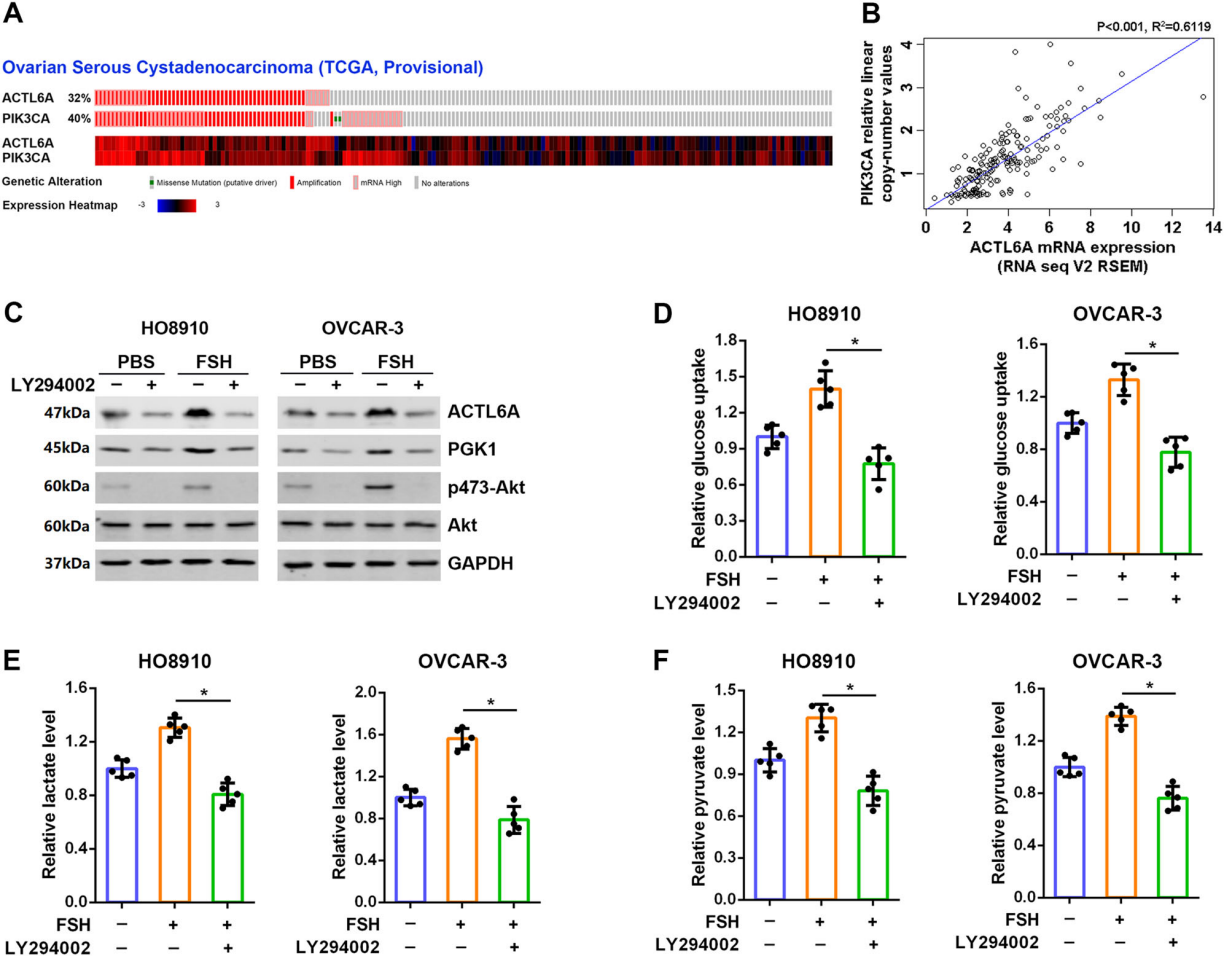
FSH upregulation of ACTL6A and PGK1 depends on PI3K/AKT signaling. **a** Genetic profiling of ACTL6A and PIK3CA co-occur in ovarian cancer from TCGA databases determined by cBioPortal analysis (http://www.cbioportal.org/). **b** Scatterplots of ACTL6A mRNA expression vs. PIK3CA copy number in ovarian cancer from TCGA databases. **c** LY294002 blocks FSH-induced ACTL6A and PGK1 expression and AKT activation. **d**–**f** Glucose uptake (**d**), lactate production (**e**), and pyruvate level (**f**) were measured in HO8910 and OVCAR-3 cells stimulated by FSH with or without LY294002 as indicated. *p < 0.01

It has been reported that ACTL6A is co-amplified with PIK3CA and SOX2 in the 3q26 amplicon in squamous cell carcinoma^18^, which is confirmed in ovarian cancer according to the TCGA database analyzed by cBioPortal (Fig. 7a and Supplementary Fig. S5a). Also there exists positive correlation between mRNA expression of ACTL6A and copy numbers of PIK3CA (*R*^2^ = 0.6119, *p* < 0.001; Fig. 7b). Moreover, ACTL6A is downregulated after treatment of the PI3K inhibitor LY294002 and the AKT antagonist AZD5363 in GSE88831 and GSE69893 databases (Supplementary Fig. S5b). Treatment of LY294002 or AKT1/2/3 inhibitor MK2206 also resulted in reduction in ACTL6A protein level (Fig. 7c and Supplementary Fig. S5c). As PI3K/AKT is believed to be one of the main signaling pathway activated by FSH, we hypothesized that FSH may regulate ACTL6A through PI3K/AKT pathway. As expected, treatment of LY294002 resulted in a reduction in ACTL6A and p-AKT protein levels; more importantly, FSH could not reverse the inhibitory effect of LY294002 on the expression of ACTL6A and PI3K/AKT activity (Fig. 7c). Similar expression patterns of PGK1 was also observed (Fig. 7c). Finally, we examined whether PI3K/AKT signaling is involved in FSH-mediated glycolysis. Indeed, additional treatment with LY294002 blocked the activation of glycolysis in cells stimulated by FSH (Fig. 7d–f). Taken together, these data indicate that PI3K/AKT activity is required for FSH-induced glycolysis and ACTL6A expression.

## Discussion

**Fig. 8:**
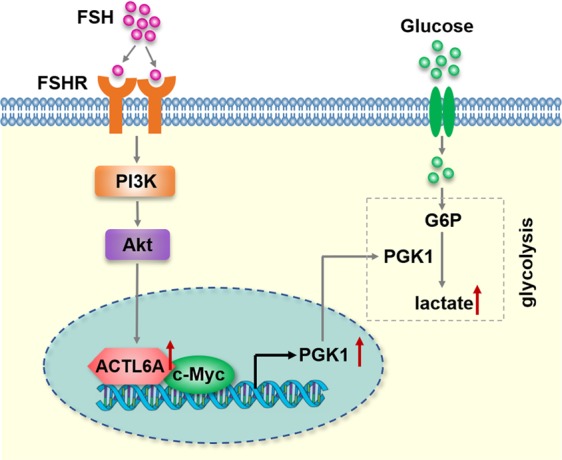
Model for Proposed Role for ACTL6A in ovarian cancer. The schematic describes a proposed model for the role of ACTL6A involved in FSH-driven glycolysis in ovarian cancer cells through upregulating PGK1

During the past decade, studies have greatly advanced our knowledge of cellular metabolism. Aerobic glycolysis, one of the main metabolic pathways, is critically involved in tumor growth and therapy resistance^4,37^. Therefore, targeting glycolysis in ovarian cancer cells may potentially be an important therapeutic strategy to improve patient survival. To our best knowledge, we uncovered a previously unreported role of ACTL6A in glycolysis. We identified the critical roles of ACTL6A and PGK1 expression in glycolysis in ovarian cancer and the novel function for ACTL6A in FSH-induced glycolysis (Fig. 8).

The function of ACTL6A in tumorigenesis has been characterized in several studies. These roles are mainly concentrated in cell proliferation, migration, invasion, tissue regeneration, and stem cell properties^16–20^. Similar to previous findings, our study suggests that ACTL6A is involved in the ovarian carcinogenesis process and promotes cell growth and motility. The genomic and expression profiling of ACTL6A in ovarian cancer has been determined based on TCGA data analysis and IHC detection. Our work also revealed a clinical correlation between ACTL6A and overall survival in two independent clinical cohorts from GEO database. In addition to these well-known functions mentioned above, we put forward evidence that ACTL6A regulates glycolysis in ovarian cancer cells. During glycolysis, glucose or glycogen is broken down into pyruvate and then converted into lactate^38^. Herein, we assessed the glucose uptake, lactate and pyruvate production in HO8910 and OVCAR-3 cells transfected with shRNA against ACTL6A. Downregulation of ACTL6A expression resulted in reduced glucose utilization, lactate production, and pyruvate levels, whereas overexpression of ACTL6A promoted them. Therefore, the current findings add new biological activities to elucidate how ACTL6A promotes tumor growth and migration.

In an effort to clarify the mechanism by which ACTL6A regulates glycolysis, we utilized TCGA and GEO databases for data mining and confirmed that PGK1 was the downstream target of ACTL6A. As an ATP-generating glycolytic enzyme identified decades ago, PGK1 has been found to be overexpressed in a variety of human tumors and to be associated with poor survival^11,12,39^. PGK1 promotes tumor cell invasion by activating the AKT and ERK pathways^40^, whereas ERK not only induces phosphorylation and mitochondrial translocation of PGK1 and pyruvate dehydrogenase kinase 1 phosphorylation^41^ but also activates casein kinase 2α to phosphorylate nuclear PGK1 at S256, phosphorylated PGK1 binds to the kinase cell division cycle 7, and converts ADP to ATP^42^. Furthermore, acetylation at K323 of PGK1 increases its enzymatic activity promotes cancer cell metabolism^11^. Besides its critical role in glycolysis, PGK1 is thought to be involved in additional molecular and cellular functions. For example, PGK1 enhances the chaperone activity of Hsp90 and promotes multi-stress resistance^43^. Moreover, PGK1 mediates DNA repair and contributes to chemoresistance and radioresistance in cancers^44–46^. Here we found that ACTL6A-mediated glycolysis in ovarian cancer cells via regulation of PGK1. Several lines of evidences support this notion. First, silencing of ACLT6A led to decreased expression of PGK1, whereas overexpression of ACTL6A had the opposite effect. Second, PGK1 expression was regulated by ACTL6A in a c-Myc-dependent manner. Third, the expression of ACLT6A was positively correlated with PGK1 in clinical samples of ovarian cancer and tumor xenografts. Thus, the present study provides mechanistic and clinical evidence supporting a model that ACTL6A induces glycolysis in ovarian cancer cells via PGK1.

Given that FSH as a risk factor for EOC development, the molecular mechanisms of FSH-induced ovarian carcinogenesis have been widely studied^47,48^. FSH binds to FSHR and activates downstream signaling including PKA, PI3K/AKT, ERK1/2, mitogen-activated protein kinase, and Notch pathways, thereby facilitating malignant cell behavior of ovarian cancer^25–27,49^. In a very recent study, Li et al.^28^ stated that FSH upregulated the expression of PKM2 and promoted glycolysis. These findings prompted us to explore whether ACTL6A is affecting the glycolysis induced by FSH. Our work provided evidence that ACTL6A is upregulated by FSH, which is dependent on PI3K/AKT signaling. We observed that knockdown of ACTL6A abrogated FSH-induced glucose utilization, lactate production, and pyruvate levels. Moreover, enhanced cell growth and migration stimulated by FSH was also suppressed after silencing of ACTL6A. Although these data suggest that targeting ACTL6A is one strategy to attenuate FSH-induced glycolysis, it is possible that additional mechanisms are involved.

To summarize, our model positions ACTL6A as a regulator of glycolysis through PGK1. Dysregulation of ACTL6A might be a novel carcinogenic process involved in FSH-induced tumorigenesis of ovarian cancer we describe here. Collectively, our data provide a new mechanism for tumorigenicity of ACTL6A and reveal a previously unappreciated role for FSH/ACTL6A/PGK1 axis facilitates glycolysis in ovarian cancer.

## Supplementary information

DECLARATION OF CONTRIBUTIONS TO ARTICLE

Supplementary Table S1

Supplementary Table S2

Supplementary Table S3

Supplementary Figure S1

Supplementary Figure S2

Supplementary Figure S3

Supplementary Figure S4

Supplementary Figure S5
