# Supplementary material for: ACTL6A regulates follicle-stimulating hormone-driven glycolysis in ovarian cancer cells via PGK1
Source: Cell Death Dis. 2019 Oct 24;10(11):811. doi: 10.1038/s41419-019-2050-y (PMC6813335; doi:10.1038/s41419-019-2050-y)
Supplement: Supplementary file 1 — DECLARATION OF CONTRIBUTIONS TO ARTICLE [file 41419_2019_2050_MOESM1_ESM.pdf]

DECLARATION OF CONTRIBUTIONS TO ARTICLE

ADMC

Manuscript Number:	Journal Name:	
CDDIS-19-2560R	*Cell Death & Differentiation*	(the 'Journal')

Proposed Title of the Contribution:

ACTL6A regulates follicle-stimulating hormone-driven glycolysis in ovarian cancer cells via PGK1 (the 'Contribution)

Author(s):

Jiawen Zhang, Jing Zhang, Yingze Wei, Qingxian Li, Qingying Wang (the 'Authors')

For all *CDD* articles, each person named as an author in the published version must be able to show he or she has contributed substantially to the article.

Authorship credit should be based on 1) substantial contributions to conception and design, acquisition of data, or analysis and interpretation of data; 2) drafting the article or revising it critically for important intellectual content; and 3) final approval of the version to be published. Authors should meet conditions 1, 2 and 3.

Any person who cannot be shown to have made a substantial contribution to the article cannot be listed as an author in the final version. The name of any person who is deemed to have made a minor contribution can, however, appear in the Acknowledgments section of the article.

Please complete the table below to indicate the contributions of all named authors to the manuscript.

Author Full Name:	Specification of Contribution to the Manuscript:
Jiawen Zhang	Conceive the project and designed experiments,perform experiments,analyze the data,write the manuscript.
Jing Zhang	Perform experiments,analyze the data.
Yingze Wei	Perform experiments,analyze the data.
Qingxian Li	Perform experiments.
Qingying Wang	Conceive the project and designed experiments,analyze the data,write the manuscript.

Please complete the table below to indicate the contributions of all named authors to the figures.

Figure 1:

Jiawen Zhang, Yingze Wei, Qingying Wang

Figure 2:

Jing Zhang, Qingxian Li

Figure 3:

Jing Zhang, Qingxian Li

Figure 4:

Jiawen Zhang, Yingze Wei, Qingying Wang

Figure 5:

Jiawen Zhang, Yingze Wei, Qingying Wang

Figure 6:

Jiawen Zhang, Jing Zhang, Qingxian Li

Signed for and on behalf of the Author(s):	Print Name:	Date:
Qingying Wang	Qingying Wang	08/10/2019
