# Supplementary material for: ACTL6A regulates follicle-stimulating hormone-driven glycolysis in ovarian cancer cells via PGK1
Source: Cell Death Dis. 2019 Oct 24;10(11):811. doi: 10.1038/s41419-019-2050-y (PMC6813335; doi:10.1038/s41419-019-2050-y)
Supplement: Supplementary file 2 — Supplementary Table S1 [file 41419_2019_2050_MOESM2_ESM.docx]

Supplementary Table S1. The correlation between the expression of ACTL6A and glycolysis-related genes in TCGA database

| Gene | Spearman's Correlation | *p* Value |
| --- | --- | --- |
| ALDOA | 0.142 | 0.0127 |
| ALDOB | -0.136 | 0.0175 |
| ALDOC | 0.027 | 0.638 |
| BPGM | 0.154 | 6.87e-03 |
| ENO1 | 0.211 | 1.96e-04 |
| ENO2 | -0.0085 | 0.882 |
| ENO3 | 5.98e-03 | 0.917 |
| GALM | 0.0329 | 0.566 |
| GCK | 0.0674 | 0.239 |
| GPI | 0.184 | 1.20e-03 |
| HK2 | -0.121 | 0.0347 |
| HK3 | 0.0259 | 0.652 |
| PFKP | 0.0393 | 0.493 |
| PFKL | -0.154 | 6.92e-03 |
| PGAM1 | 0.184 | 1.24e-03 |
| PGAM2 | 0.03 | 0.601 |
| PGK1 | 0.312 | 2.47e-08 |
| PGK2 | 0.1 | 0.0793 |
| PGM1 | 0.0516 | 0.368 |
| PGM2 | 0.0906 | 0.113 |
| PGM3 | 0.213 | 1.73e-04 |
| PKLR | 0.0815 | 0.154 |
| PKM2 | 0.0925 | 0.106 |
| LDHA | 0.109 | 0.0556 |
| LDHB | 0.0967 | 0.0909 |
| LDHC | 0.101 | 0.0763 |
| PDK1 | 0.162 | 4.35e-03 |
| GOT1 | 0.184 | 1.21e-03 |
| GOT2 | 0.0898 | 0.116 |
| PFKFB1 | 0.202 | 3.80e-04 |
| PFKFB2 | -0.102 | 0.0747 |
| PFKFB3 | -0.136 | 0.0172 |
| PFKFB4 | -0.00551 | 0.923 |
