# Supplementary material for: ACTL6A regulates follicle-stimulating hormone-driven glycolysis in ovarian cancer cells via PGK1
Source: Cell Death Dis. 2019 Oct 24;10(11):811. doi: 10.1038/s41419-019-2050-y (PMC6813335; doi:10.1038/s41419-019-2050-y)
Supplement: Supplementary file 3 — Supplementary Table S2 [file 41419_2019_2050_MOESM3_ESM.docx]

Supplementary Table S2. The alterations of glycolysis-related genes in GSE88831

| Gene | ID | Adj. *p* Value | *p* Value |
| --- | --- | --- | --- |
| ALDOA | 7994737 | 0.57923 | 0.276 |
| ALDOB | 8162884 | 0.90136 | 0.784 |
| ALDOC | 8013660 | 0.81401 | 0.62 |
| BPGM | 8136341 | 0.41923 | 1.19e-01 |
| ENO1 | 7912198 | 0.02744 | 4.64e-04 |
| ENO2 | 7953532 | 0.14563 | 1.07e-02 |
| ENO3 | 8004043 | 0.35007 | 7.13e-02 |
| GALM | 8041542 | 0.71481 | 0.459 |
| GCK | 8139314 | 0.89164 | 0.766 |
| GPI | 8027621 | 0.45736 | 0.149 |
| HK2 | 8042942 | 0.51179 | 0.201 |
| HK3 | 8115957 | 0.44143 | 0.135 |
| PFKP | 7925876 | 0.52756 | 0.218 |
| PFKL | 8069057 | 0.97741 | 0.946 |
| PGAM1 | 7929624 | 0.29777 | 4.56e-02 |
| PGAM2 | 8139276 | 0.45397 | 0.146 |
| PGK1 | 8168500 | 0.03797 | 9.26e-04 |
| PGK2 | 8126916 | 0.31191 | 5.16e-02 |
| PGM1 | 7901951 | 0.56035 | 0.254 |
| PGM2 | 8094556 | 0.60128 | 0.304 |
| PGM3 | 8127841 | 0.0533 | 1.73e-03 |
| PKLR | 7920744 | 0.72035 | 0.469 |
| PKM2 | 7990151 | 0.66667 | 0.39 |
| LDHA | 7938777 | 0.1833 | 1.66e-02 |
| LDHB | 7961693 | 0.44935 | 0.142 |
| LDHC | 7938788 | 0.7392 | 0.497 |
| PDK1 | 8046408 | 0.4108 | 1.12e-01 |
| GOT1 | 7935627 | 0.39679 | 1.00e-01 |
| GOT2 | 8001764 | 0.26242 | 3.43e-02 |
| PFKFB1 | 8173120 | 0.96375 | 0.915 |
| PFKFB2 | 7909285 | 0.16763 | 1.40e-02 |
| PFKFB3 | 7926037 | 0.20882 | 2.13e-02 |
| PFKFB4 | 8086961 | 0.99476 | 0.986 |
