# Supplementary material for: ACTL6A regulates follicle-stimulating hormone-driven glycolysis in ovarian cancer cells via PGK1
Source: Cell Death Dis. 2019 Oct 24;10(11):811. doi: 10.1038/s41419-019-2050-y (PMC6813335; doi:10.1038/s41419-019-2050-y)
Supplement: Supplementary file 4 — Supplementary Table S3 [file 41419_2019_2050_MOESM4_ESM.docx]

Supplementary Table S3. The sequences for shRNA and siRNA used in this study.

| shRNA / siRNA | Oligomers (5’-3’) |
| --- | --- |
| ACTL6A | CGGTACTTCAAGTGTCAGATT |
| PGK1 | GCTTCTGGGAACAAGGTTAAA |
| c-Myc | CCCAAGGTAGTTATCCTTAAA |
| FSHR | CCTAACTACCAGCCAATATAA |
