# Supplementary figures and images for: ACTL6A regulates follicle-stimulating hormone-driven glycolysis in ovarian cancer cells via PGK1
Source: Cell Death Dis. 2019 Oct 24;10(11):811. doi: 10.1038/s41419-019-2050-y (PMC6813335; doi:10.1038/s41419-019-2050-y)

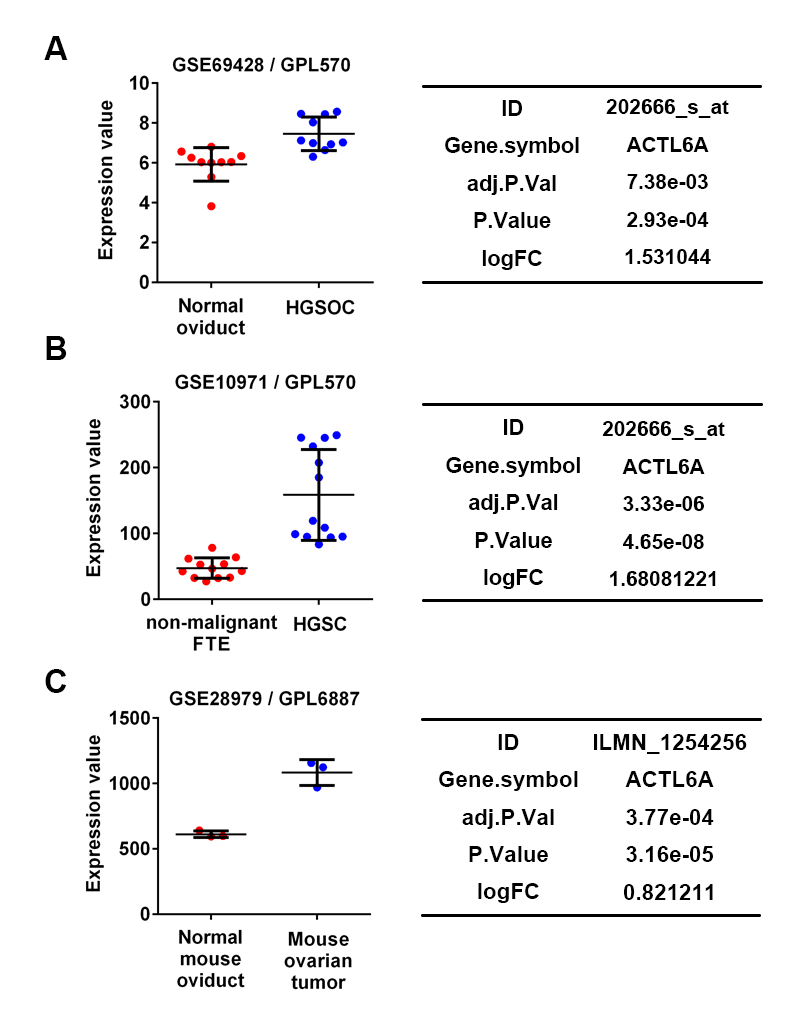

Supplement: Supplementary file 5 — Supplementary Figure S1 [file 41419_2019_2050_MOESM5_ESM.tif]

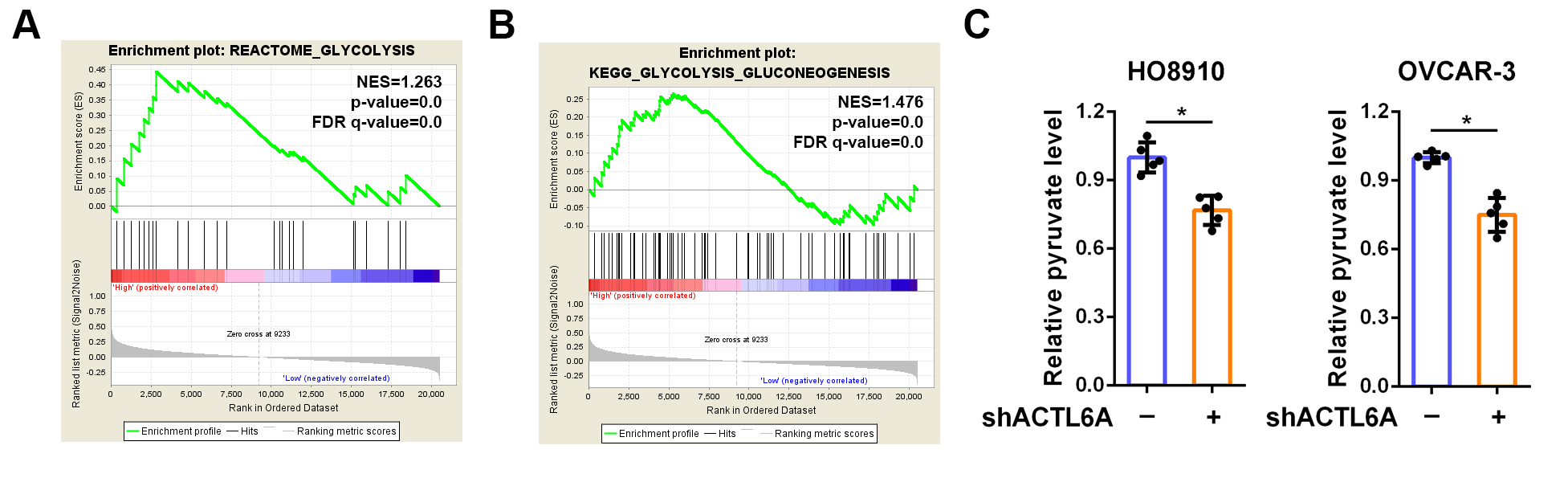

Supplement: Supplementary file 6 — Supplementary Figure S2 [file 41419_2019_2050_MOESM6_ESM.tif]

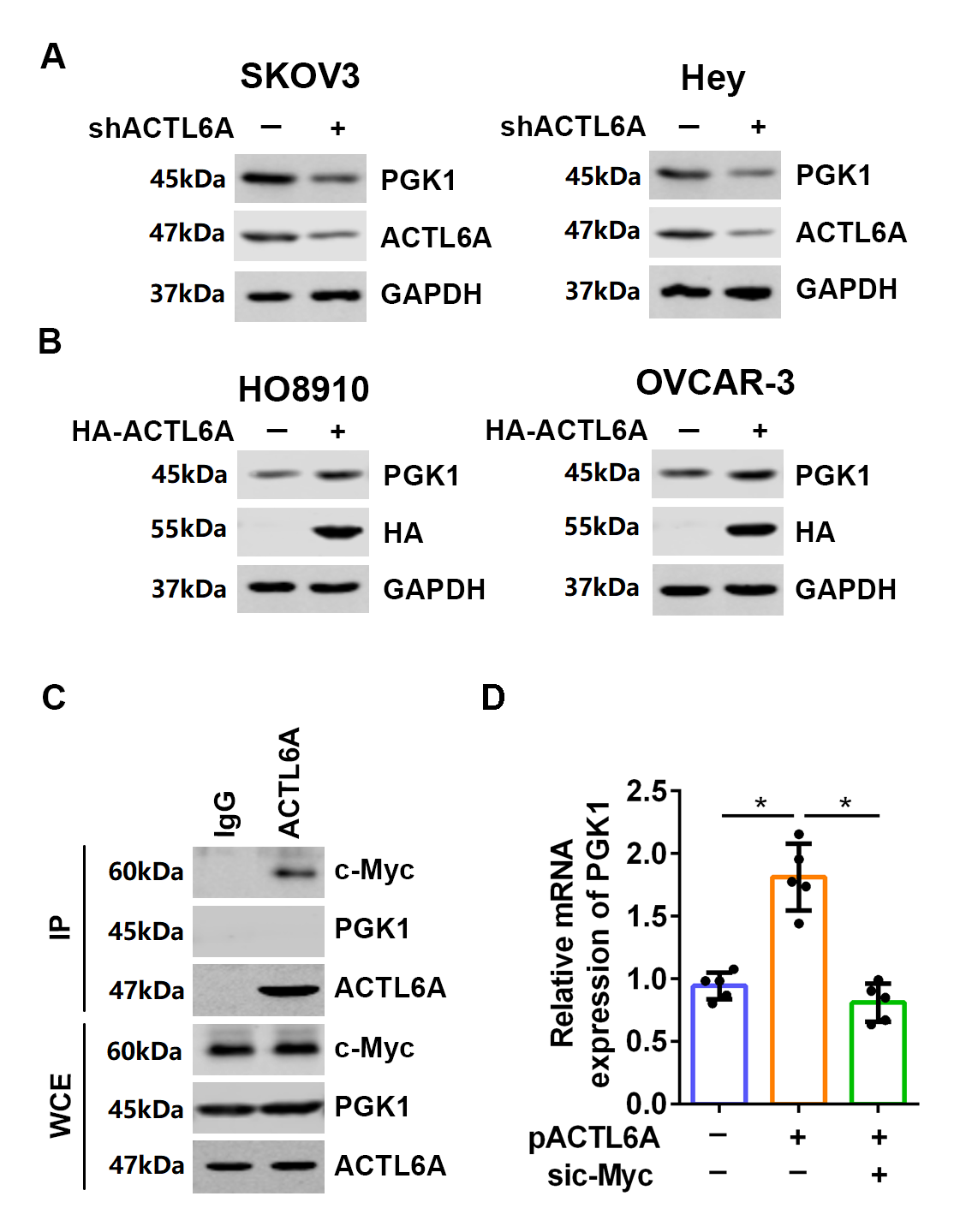

Supplement: Supplementary file 7 — Supplementary Figure S3 [file 41419_2019_2050_MOESM7_ESM.tif]

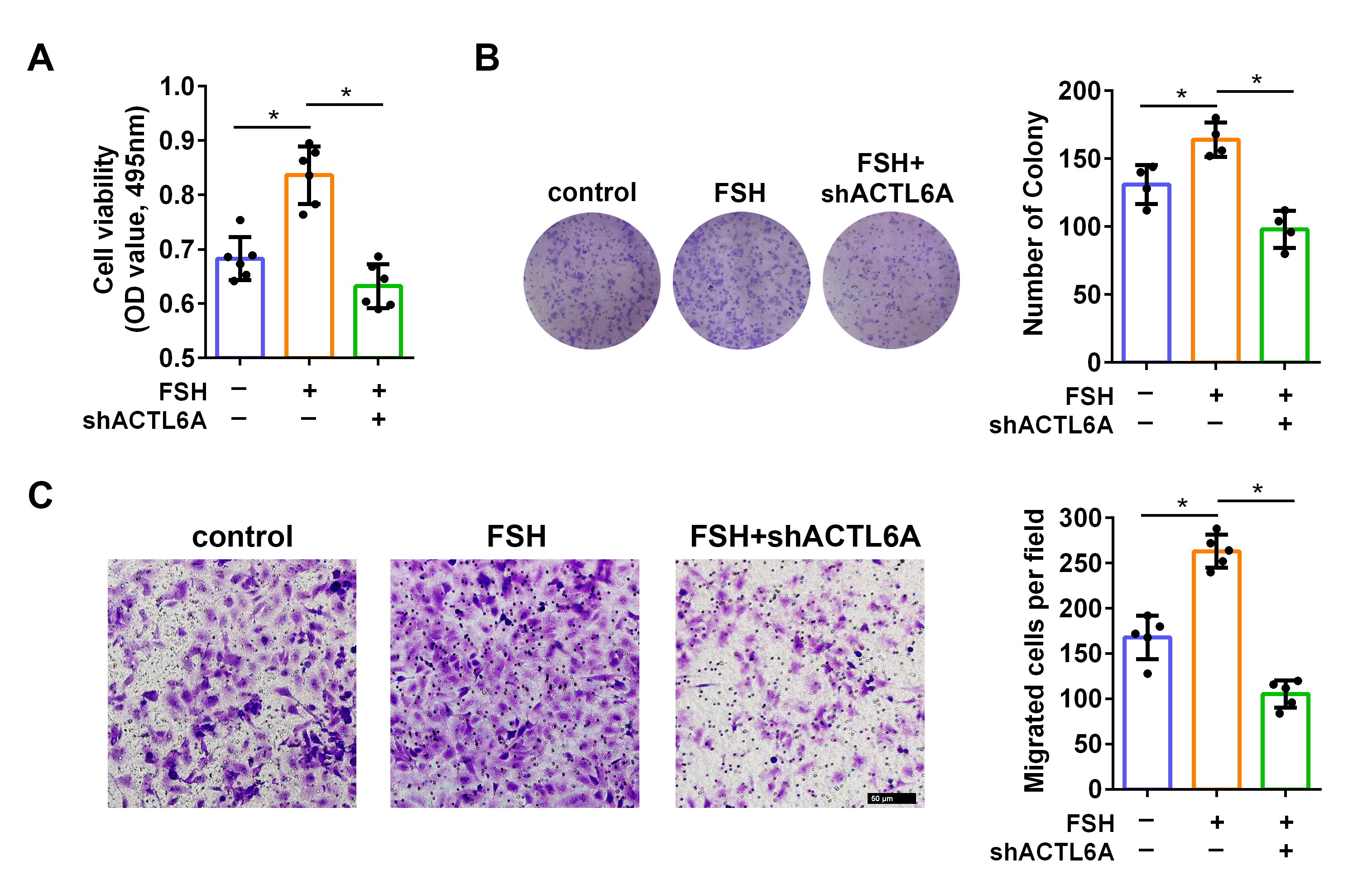

Supplement: Supplementary file 8 — Supplementary Figure S4 [file 41419_2019_2050_MOESM8_ESM.tif]

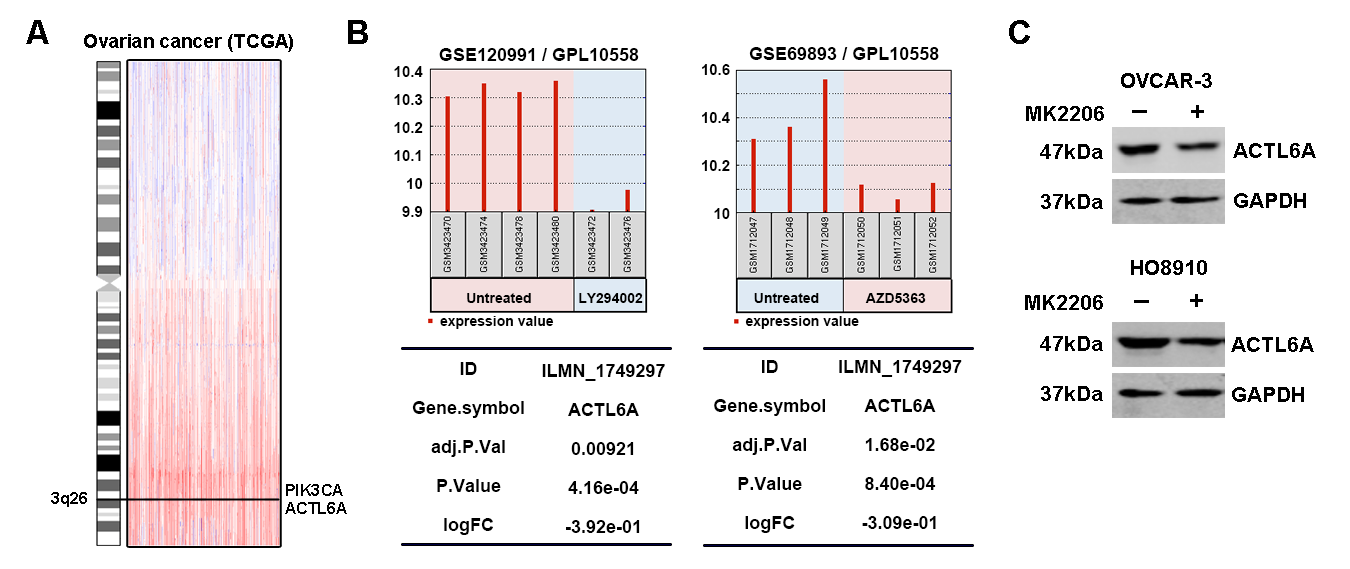

Supplement: Supplementary file 9 — Supplementary Figure S5 [file 41419_2019_2050_MOESM9_ESM.tif]
